# Influence of Buffers, Ionic Strength, and pH on the Volume Phase Transition Behavior of Acrylamide-Based Nanogels

**DOI:** 10.3390/polym12112590

**Published:** 2020-11-04

**Authors:** Harriet Louise Judah, Pengfei Liu, Ali Zarbakhsh, Marina Resmini

**Affiliations:** Department of Chemistry, SBCS, Queen Mary University of London, Mile End Road, London E1 4NS, UK; h.l.judah@se15.qmul.ac.uk (H.L.J.); pengfei.liu@qmul.ac.uk (P.L.)

**Keywords:** thermoresponsive, crosslinked nanogel, functional monomers, hydrogels, NIPAM, ionic strength

## Abstract

The use of covalently crosslinked nanogels for applications in biology and medicine is dependent on their properties and characteristics, which often change because of the biological media involved. Understanding the role of salts, ionic strength and pH in altering specific properties is key to progress in this area. We studied the effect of both chemical structure and media environment on the thermoresponsive behavior of nanogels. A small library of methylenebisacrylamide (MBA) crosslinked nanogels were prepared using *N*-isopropylacrylamide (NIPAM) or *N-n*-propylacrylamide (NPAM), in combination with functional monomers *N*-hydroxyethylacrylamide (HEAM) and *N*-acryloyl-l-proline (APrOH). The thermoresponsive properties of nanogels were evaluated in phosphate buffer, tris-acetate buffer and Ringer HEPES, with varying concentrations and ionic strengths. The presence of ions facilitates the phase separation of nanogels, and this “salting-out” effect strongly depends on the electrolyte concentration as well as the specificity of individual anions, e.g., their positions in the Hofmeister series. A subtle change in the chemical structure of the side chain of the monomer from NIPAM to NPAM leads to a reduction of the volume phase transition temperature (VPTT) value by ~10 °C. The addition of hydrophilic comonomers such as HEAM, on the other hand, causes a ~20 °C shift in VPTT to higher values. The data highlight the significant role played by the chemical structure of the monomers used, with hydrophobicity and rigidity closely interlinked in determining thermoresponsive behavior. Furthermore, the volume phase transition temperature (VPTT) of nanogels copolymerized with ionizable APrOH comonomer can be tailored by changes in the pH of buffer solutions. This temperature-controlled phase transition is driven by intricate interplay involving the entropy of mixing, electrostatic interactions, conformational transitions, and structural rigidity. These results highlight the importance of understanding the physiochemical properties and behavior of covalently crosslinked nanogels in a biological environment prior to their applications in life-science, such as temperature/pH-triggered drug delivery systems.

## 1. Introduction

Nanogels are interesting materials that are being studied for applications in varied areas, such as catalysis, sensing and drug delivery [1,2,3,4,5,6,7], to cite a few. Recent studies have demonstrated the potential of these nanoparticles in nanomedicine [8,9,10] due to their nanoscale size, with a high surface-to-volume ratio and the ability to form thermodynamically stable colloidal dispersions. While nanogels are inherently soft in character, covalent crosslinking of polymer chains yields a three-dimensional network that maintains structural integrity [11,12,13], with the degree of rigidity dependent on crosslinker content. Moreover, these materials are highly versatile and can be tailored to possess specific surface functionalities by including different monomers in the formulation [14,15,16,17]. Such changes in the formulation also allow the introduction of stimuli-responsive properties, such as pH [18], temperature [19], magnetic field [20], ionic force [21] or a combination thereof [22,23]. Among these, temperature is the most widely studied stimulus due to its physiological importance. Thermoresponsive nanogels experience conformational changes in response to temperature, leading to changes in morphology such as size, shape and water solubility, properties that are fundamentally dictated by the interplay between hydrophilicity and hydrophobicity [24,25].

Furthermore, applications of thermoresponsive nanogels often involve their interaction with a variety of surfaces and in different solutions. It has been demonstrated that polymer formulations can influence thermoresponsive properties and also their interfacial behavior [26]. Biological media such as blood contain a multitude of salts and proteins [27], which are known to influence properties due to the formation of noncovalent interactions [28] and biomolecular corona [29]. This results in changes in hydrodynamic size and surface charge, potentially leading to aggregation. For this reason, it is critical to understand how salts, pH and ionic strength can influence properties in order to tailor the formulation to the desired application.

*N*-isopropylacrylamide (NIPAM)-based polymers are known to respond to changes in temperature by undergoing conformational change [30,31,32]. When the polymers are linear, this transition is identified by the lower critical solution temperature (LCST) and for poly(NIPAM) is around 32 °C, while the introduction of the covalent crosslinker changes the structure and the conformational change is identified by the volume phase transition temperature (VPTT) [33]. 

A number of groups [34,35] have demonstrated the effect of salts and other matrices on the transition temperature of NIPAM polymers, exploiting optical and spectroscopic techniques to do so. In particular, Taha et al. [36] looked at the interaction of biological buffers with poly(NIPAM), confirming that buffers decreased the LCST values of poly(NIPAM) aqueous solutions (much like Hoffmeister ions) and originated from changes in the polymer’s hydration shell. In the work by Cremer et al. [37,38], the authors investigated the mechanism of aggregation and precipitation of poly(NIPAM) in the presence of various anions. The work demonstrated three direct interactions, polarization, surface tension and direct binding, in which the anions can affect the transition and precipitation behavior of poly(NIPAM) macromolecules. In the recent work by Drummond et al. [39,40,41], the effect of different organic and inorganic ions from the extended Hofmeister series on the solubility of temperature-sensitive NIPAM polymers was systematically studied. Their results were interpreted by the accumulation/exclusion mechanisms of ions at the interface between the NIPAM network and aqueous medium, revealing that the affinity among solvents, ions and interfaces was essential to explain the ionic specificity. However, most of these studies focused on the interaction of ions with linear poly(NIPAM) chains or NIPAM-based microgels with a fixed content of crosslinkers. The rigidity of these materials is influenced by the percentage of crosslinker incorporated, which in turn has a significant impact on their morphology and physicochemical properties, as we recently reported [14]. Consistent trends between chemical structure, particle size and VPTT were identified. In addition, dual pH and temperature-responsive nanogels based on NPAM and l-proline were prepared, and their ability to release cargo was demonstrated using Nile Blue A as a model drug. More recently, we also conducted an NMR investigation of how monomers with different reactivities are incorporated into the nanogel’s structure [42]. This is of particular importance due to the random nature of polymerization, emphasizing the need to have sound knowledge of nanogel composition to draw accurate conclusions. Reaction dynamics were probed further by altering experimental parameters such as temperature, reaction time and initiator concentration. It was shown that the addition of comonomers alters reaction kinetics, thus influencing the final composition. However, a detailed correlation between crosslinker content, thermoresponsive behavior and changes in the environmental media have yet to be reported and this has been the focus of this work. In addition, the influence of biologically relevant media on their phase transition is not as thoroughly investigated. This is rather counterintuitive considering the medically oriented applications that are proposed in the literature.

Here, we report the results of our studies on the impact that salts, ionic strength and pH can have on the properties of covalently crosslinked nanogels, and whether the degree of crosslinking and the structure of functional monomers also play a role. We used surfactant-free high dilution radical polymerization to obtain a small library of MBA-crosslinked nanogels (5 to 20%). NIPAM and NPAM-based nanogels were used with functional monomers *N*-hydroxyethyl acrylamide (HEAM) or l-proline to increase hydrophilicity. Thermoresponsive properties were studied using a selection of aqueous buffers to identify trends and specific effects.

## 2. Materials and Methods

### 2.1. Materials

All materials were used without further purification unless stated otherwise. *N*-isopropylacrylamide (NIPAM), propylamine, *N*-hydroxyethylacrylamide (HEAM), *N*,*N*′-methylenebisacrylamide (MBA), bis(2-hydroxyethyl)amino-tris(hydroxymethyl)methane (BIS-TRIS), 1,2,4,5-tetramethylbenzene (TMB) and anhydrous dimethylsulfoxide were all purchased from Sigma-Aldrich (Gillingham, UK). 2,2′-azobisisobutyronitrile (AIBN) was purchased from Sigma and recrystallized in methanol. Acryloyl chloride was purchased from Alfa Aesar (Heysham, UK). Sodium chloride was purchased from Fisher Scientific (Loughborough, UK). Glucose was purchased from Fluorochem (Hadfield, UK). Tris(hydroxymethyl)aminomethane (TRIS), hydrochloric acid (37%), sodium acetate trihydrate, sodium dihydrogen phosphate monohydrate sodium phosphate dibasic, potassium chloride, calcium chloride, magnesium chloride, sodium bicarbonate and 4-(2-hydroxyethyl)piperazine-1-ethanesulfonic acid (HEPES) were purchased from VWR Chemicals (Lutterworth, UK). *N-n*-propylacrylamide (NPAM) and *N*-acryloyl-*l*-proline (APrOH) were synthesized following a previously reported procedure [14]. Cellulose dialysis membrane (molecular weight cut-off: 3500 Da, width: 34 mm, diameter: 22 mm) was purchased from Medicell International Ltd. (London, UK).

### 2.2. Synthesis of Nanogels

Nanogels were synthesized by high dilution radical polymerization (HDRP) according to our previously reported procedure [43]. Monomers (NIPAM or NPAM), comonomers (HEAM or APrOH) and MBA as a covalent crosslinker (XL) in a variety of monomer/XL ratios were dissolved in anhydrous DMSO in a Wheaton™ bottle. The volume of solvent used was fixed to give a monomer concentration (C_m_) of 1% (*w*/*w*). AIBN (1 mol % of total moles of double bonds) was added as the initiator. The vessel was sealed, purged with N_2_ and heated to 70 °C for 24 h. The resulting nanogel solution was dialyzed against deionized water for 3 days, changing the water periodically to remove unreacted material and solvent. Finally, the polymer was isolated by freezing in liquid N_2_ and lyophilization (LTE Scientific Lyotrap), yielding a white powder that was stored dry at room temperature.

### 2.3. Preparation of Buffers

Phosphate buffer (PB), tris-acetate buffer (TAB) and Ringer HEPES buffer (RHB) were prepared according to the buffer recipes tabulated in Table S1. Ionic strength (I) is a measure of the electric field strength within a solution and was calculated for each buffer using Equation (1).

(1) $$\begin{array}{c} I=\frac{1}{2}\;\times \;\sum c_{i}z_{i}{}^{2} \end{array}$$

where c_i_ is the molarity of ion i and z_i_ is the charge number of that ion. For the weak conjugated acid/base components in the buffer recipe, their concentration in buffer media was estimated via the Henderson–Hasselbalch equation using pKa values at 25 °C.

(2) $$\begin{array}{c} pH=\;pK_{a}+\log_{10}\frac{\left[base\right]}{\left[acid\right]} \end{array}$$

### 2.4. Procedure for the Determination of the Volume Phase Transition Temperature

VPTT measurements of all nanogels in this study were carried out by measuring the light transmittance (at 500 nm) of filtered (0.20 μm pore size) nanogel solutions as a function of temperature from 20 to 65 °C at a rate of 0.5 °C min^−1^ using a Cary 100 UV–Visible Spectrophotometer (Agilent Technologies, Santa Clara, CA, USA), coupled with temperature controller. All nanogels were prepared at a concentration of 1.0 mg/mL in either deionized water, PB, TAB or RHB. The VPTT value was determined as follows: (i) the first-order derivative of the percentage of transmittance against temperature was plotted. (ii) It was then fitted with the Gaussian peak function using the Origin Lab software to find the point of inflection. (iii) The *x*-axis of this point was then taken as the VPTT value.

### 2.5. Dynamic Light Scattering

Nanogels hydrodynamic diameter measurements were obtained by dynamic light scattering (DLS) with a Zetasizer Nano ZS (Malvern Instruments Ltd., UK) at 20 °C (in some cases down to 15 °C). Suspensions of nanogel in each medium were prepared at a concentration of 1.0 mg/mL, sonicated for 10 min and filtered through a 0.20 µm filter prior to measurements. All measurements were performed in triplicate. Size distribution of nanogels is given by intensity, volume and number for all nanogels.

## 3. Results and Discussion

**Figure 1:**
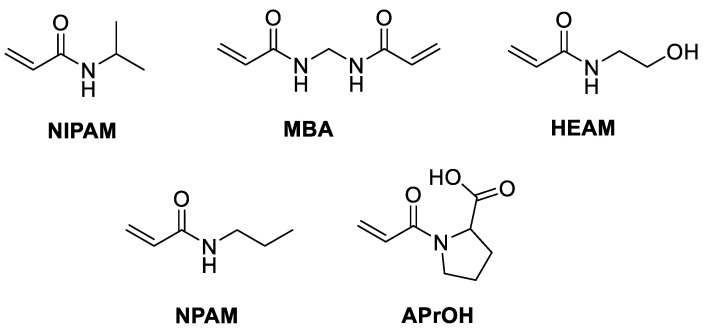
Monomers and crosslinkers used in the preparation of nanogels.

We prepared a small library of 10 nanogels synthesized using NIPAM or NPAM as the backbone monomer and MBA as the crosslinker, together with different functional monomers (shown in Figure 1 and Table 1), designed to confer a variety of surface chemistry to the nanoparticles. For each combination of functional monomers, nanogels were prepared with crosslinker content ranging from 5 to 20% to study how the matrix’s rigidity could influence the environment’s effect on thermoresponsive behavior. For this purpose, we carried out experiments first in deionized water, followed by phosphate buffer (PB, I = 25 mM, pH = 7.4), tris-acetate buffer (TAB, I = 11–30 mM, pH = 4, 6, 7.4 or 9) and ringer HEPES buffer (RHB, I = 168 mM, pH = 7.4), focusing on the effect of salts and pH in the physiological range. The specific buffers’ formulation is presented in Table S1. RHB was chosen because it is a complex matrix containing a combination of different salts and is commonly used in cell culture, effectively mimicking the biological environment. Nanogels NG1–NG10 were obtained with a >80% monomer conversion as determined by NMR [42], with a >80% chemical yield and an average particle size of ~5 nm, as determined by DLS in deionized water and in the different buffer solutions (Figures S1–S4).

### 3.1. Thermoresponsive Behavior of NIPAM–MBA Nanogels in Different Buffers

**Figure 2:**
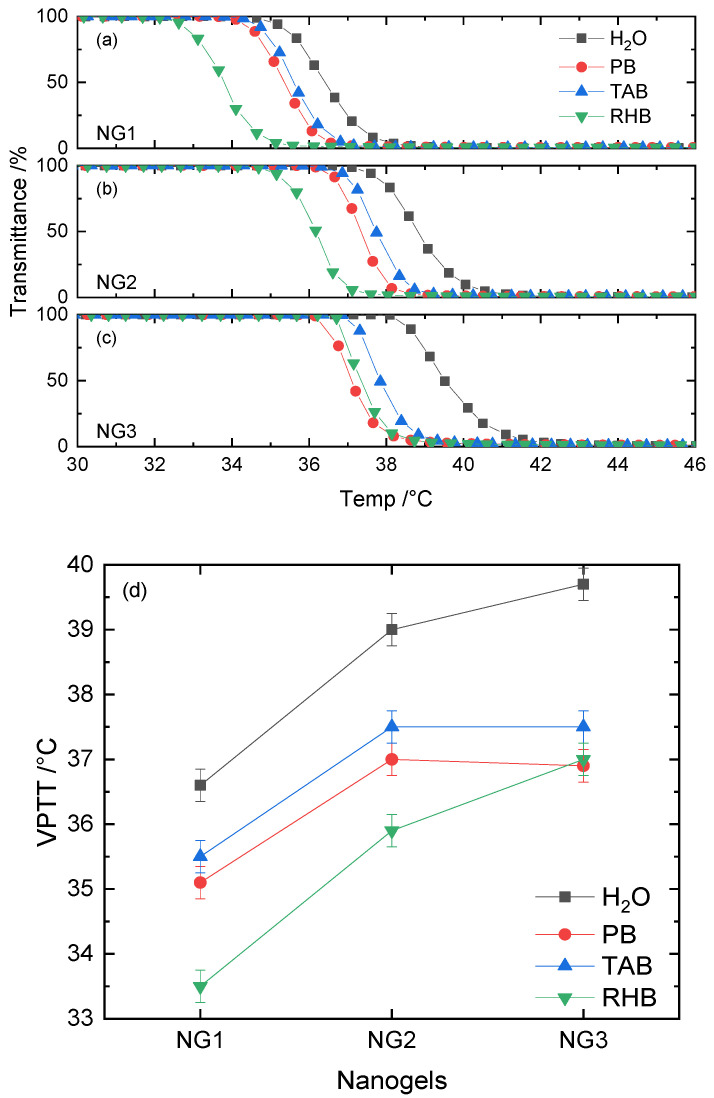
Volume phase transition temperature (VPTT) profiles of (**a**) NG1, (**b**) NG2 and (**c**) NG3 in H_2_O, phosphate buffer (PB), tris-acetate buffer (TAB) and Ringer HEPES buffer (RHB). Their corresponding VPTT values are summarized in (**d**).

Neutral NIPAM nanogels crosslinked with 5, 10 and 20% MBA were characterized first, and their data were used as a benchmark. Values of VPTT for these nanogels were evaluated first in deionized water and then in PB, TAB and RHB at pH = 7.4. Although these buffers present the same pH value, their salt concentration differs significantly (see Table S1). The ionic strength of RHB is nearly seven-fold higher than the value for PB. The VPTT profiles obtained for each nanogel are summarized in Figure 2a–c. The first-order derivative of the percentage of transmittance against temperature, demonstrating the instantaneous rate of change, was calculated and is shown accordingly in Figure S5. This instantaneous rate of change plotted as a function of temperature can be fitted by the Gaussian peak function (solid line in Figure S5) and the peak value is taken as the VPTT for each nanogel (Table S2). Results for NG1–NG3 in deionized water, PB, TAB and RHB are plotted in Figure 2d.

For all three nanogels studied, the VPTT in deionized water was found to be the highest compared to the results obtained in buffered media. In general, the transition temperature in buffers exhibited a decreasing trend (VPTT_TAB_ > VPTT_PB_ > VPTT_RHB_), with the exception of 20% crosslinked nanogels, in which case VPTT_PB_ = VPTT_RHB_ = 37 °C. When varying crosslinker content from 5 to 10%, an increase in VPTT by 2–3 °C was observed in all media. Interestingly, as the percentage of MBA doubled to 20%, there was an increase of ~1 °C in RHB, while VPTT was relatively unaffected in H_2_O, TAB and PB.

The percentage of crosslinker incorporated into the NIPAM nanogel determines the degree of rigidity as well as the hydrophobic character of the polymer. Higher percentages of MBA yield a more rigid matrix but a less hydrophobic material due to the lower concentration of isopropyl groups. In comparison to softer nanogels, rigid ones experience less conformational changes in solution upon heating, resulting in an increaase in VPTT values. When the percentage of the crosslinker is below 10%, the nanogel’s matrix is not rigid enough and the hydrophobicity factor dominates. This explains a 2 °C increase in VPTT as the percentage of MBA is increased to 10%. However, the rigidity factor dominates when the crosslinker content is further doubled to 20%, which counteracts against the hydrophobic effect. It is this interplay between hydrophobicity and rigidity of the polymer that determines the trends observed in VPTT as a function of crosslinker. Interestingly, a ~1 °C increase was still observed in RHB when the percentage of MBA doubled to 20%. This suggests the presence of a large number of inorganic ions (the ionic strength I = 168 mM) may promote the influence of the hydrophobicity interaction on nanogels’ phase separation behavior.

The presence of salts in the solution facilitates the phase separation of nanogels, which explains why the VPTT is higher in deionized water than in buffers. This behavior is attributed to the reduction in solubility of nanogels induced by the presence of dissolved ions. The hydration of ions, particularly anions, competes for water molecules, which are supposed to be bound to nanogels’ hydrophilic and hydrophobic domains. This disruption in hydration of the colloidal particles (so-called “salting-out” phenomenon) strongly depends on the electrolyte concentration as well as the specificity of individual anions (their positions in the Hofmeister series). The ionic strength of RHB is nearly seven-fold higher than that of PB and TAB, leading to significantly more intense competition between ions and polymeric entities for water molecules. Consequently, the phase transition occurs at relatively lower temperatures, i.e., lower VPTT in RHB. According to the Hofmeister sequence, the hydration strength of HPO_4_^2−^ is greater than that of CH_3_COO^−^. This justifies why the temperature required to dehydrate the nanogel particles is lower in PB than TAB, despite the similarity in ionic strength. Interestingly, the increasing rigidity of the nanogel appears to weaken the hydration disruption in the case of RHB, as the VPTT continues to increase from 10 to 20% of crosslinker. This could be due to the nanogel possessing enough rigid character to resist salting-out until a higher temperature is achieved. Having demonstrated the impact on VPTT of changing crosslinker content and also buffers, the next step focused on studying how the incorporation of functional monomers with specific chemical functionalities could further influence the properties of the nanogels.

### 3.2. NIPAM–HEAM–MBA Nanogels in Different Buffers

**Figure 3:**
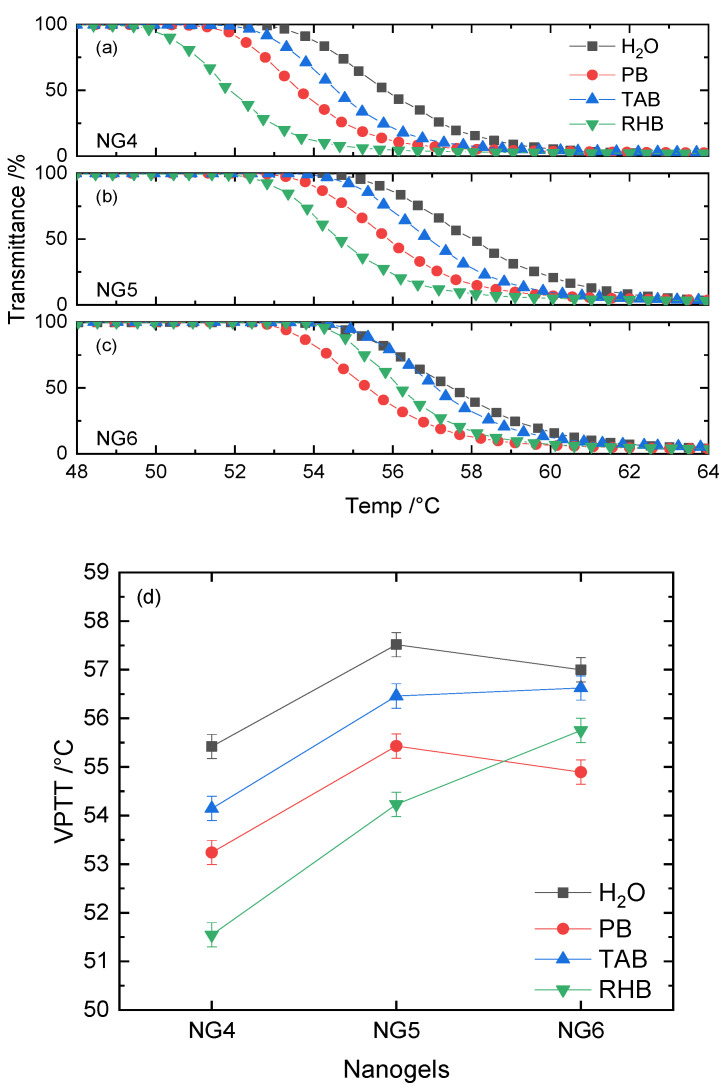
VPTT profiles of (**a**) NG4, (**b**) NG5 and (**c**) NG6 in H_2_O, PB, TAB and RHB. Their corresponding VPTT values are summarized in (**d**).

The incorporation of functional monomers alters nanogels’ morphology and physicochemical properties, providing specific functionalities for targeted applications. The first functional monomer to be considered was HEAM, chosen to introduce hydroxyl groups in the nanogel matrix and enhance the hydrogen bond capabilities. For the purpose of this study, it was decided to add 20% of the functional monomer to ensure a significant change in properties. NIPAM was retained as the backbone monomer, while the crosslinker MBA was varied from 5 to 20%. Three new nanogel preparations were obtained, with chemical yields and sizes similar to what was previously obtained. The thermoresponsive behavior was evaluated in deionized water, PB, TAB and RHB and the results obtained are summarized in Figure 3 and Figure S6 (Gaussian fits) and Table S2 (VPTT values).

For all three nanogel preparations, the data clearly highlight the significant impact of having added the HEAM monomer, with all VPTT values having increased by 18–19 °C compared to the corresponding nanogels without functional monomer. It is interesting to observe that the VPTT profiles of NG4–NG6 display a much broader transition, consistent with having a large number of hydroxyl groups on the surface. Analogous to observations made for the NIPAM–MBA nanogels, the VPTT of HEAM-containing nanogels saw a 2–3 °C increase in all media when the crosslinker content doubled from 5 to 10%, while the change from 10 to 20% crosslinker did not induce such significant change. For both NG4 and NG5, the change in environment from water to buffers with increased salt concentration resulted in a significant drop in VPTT. 

The interspersion of HEAM into the nanogel’s skeleton significantly enhances the hydrophilic character of these particles. As a result, more energy is required to break the robust hydration strength of hydroxyl groups with H_2_O, resulting in higher VPTT values. The broad phase transition of these nanogels may be ascribed to a less cooperative collapse of chain segments. The introduction of the HEAM comonomer increases the size distribution heterogeneity of the chains, thus, resulting in a less sharp transition profile.

The presence of nonionizable hydroxyl groups in the nanogel latex does not change the Hoffmeister “salting-out” effect, and for the 5% and 10% crosslinked nanogels, their sequences remain the same. However, as the crosslinker content doubles to 20%, the VPTT in TAB and RHB appears to increase, while in deionized water and PB it does not. This phenomenon may be attributed to the hydroxyl groups of the organic components in these buffers. It is possible that they are able to further stabilize NIPAM–HEAM nanogel particles via additional hydrogen bonding of hydroxyl groups with HEAM units, coupled with the densely crosslinked core of the nanogel becoming more rigid. The results highlighted that the NIPAM-based nanogels when modified with functional monomers that introduce a higher degree of hydrophilicity, result in materials with VPTT too high for most biological applications. The focus therefore moved towards changing the backbone monomer to tailor the VPTT to lower values.

### 3.3. NPAM–APrOH–MBA Nanogels in Different Buffers

**Figure 4:**
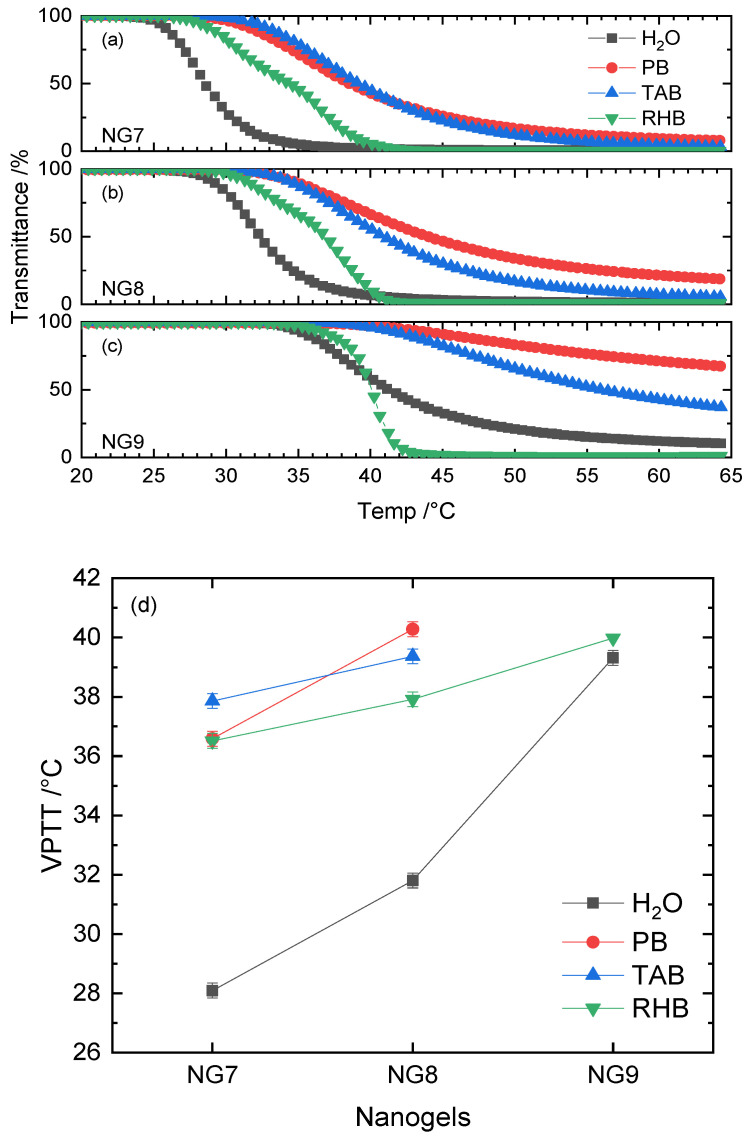
VPTT profiles of (**a**) NG7, (**b**) NG8 and (**c**) NG9 in H_2_O, PB, TAB and RHB. Their corresponding VPTT values are summarized in (**d**).

Recently, we reported that the use of *N*-*n*-propylacrylamide (NPAM) with an MBA content ranging from 10 to 50% resulted in nanogels with VPTT values 7–10 °C lower compared to the corresponding nanogels made with NIPAM. Furthermore, when NPAM–MBA was copolymerized with an *l*-proline-based monomer (APrOH), it was shown to have promising drug delivery capabilities using Nile Blue A as a drug model [14]. In light of this, we decided to change the backbone monomer to NPAM and include APrOH (2.5%) as a functional monomer; the crosslinker MBA was varied between 5, 10 and 20% to retain consistency with the previous data. VPTT measurements were conducted in deionized water, PB, TAB and RHB and the results are presented in Figure 4 and Figure S7 (Gaussian fits) and Table S2 (VPTT values).

NPAM–APrOH nanogels crosslinked with 5%MBA phase-separated in deionized water at 28 °C. As the percentage of MBA was doubled, VPTT increased by almost 4 °C. In the case of NG9, where 20%MBA was incorporated, the phase transition became much less sharp and the optical transmittance did not reach zero at higher temperatures. The VPTT of NG9 in deionized water was estimated to be slightly below 39 °C. Upon buffering of these negatively charged nanogels to pH = 7.4 using PB and TAB, the collapse and subsequent phase separation took place at approximately 10 °C higher than that of deionized water. However, the considerably shallow slopes of VPTT profiles were observed to be independent of the nanogel networks’ rigidity. For NG9 in PB and TAB, the change of transmittance is in fact far too shallow and does not allow an accurate estimate of the point of inflection (Figure S7). The VPTT profiles of NG7–NG9 in RHB are much sharper than those in both PB and TAB, although they have the same pH value (e.g., a similar degree of deprotonation of carboxylic groups in theory). Interestingly, the phase transition temperature of NG9 in RHB matches with that in deionized water.

The monomer NPAM is a linear isomer of NIPAM; therefore, its use results in an overall increase of hydrophobicity of the resultant nanogels. For this reason, lower VPTT values for NPAM over NIPAM-based nanogels are expected. The copolymerization of the functional monomer APrOH provides the nanogel with a negative charge. By suspending NPAM–APrOH nanogels in deionized water, a weakly acidic solution (around pH = 5) is obtained due to the partial ionization of carboxylic acid units. In addition to the hydrophobicity and flexibility contribution, the phase transition mechanism of these anionic nanogels is mainly governed by electrostatic interactions. We have recently reported the monomer conversion and polymerization kinetics of NPAM–APrOH–MBA nanogels [42]. It was found that this functional comonomer was highly active, having more than 95% conversion in the first two hours of the polymerization. This means the comonomer APrOH was mostly incorporated into the dense core of the nanogels. As a result, the repulsive electrostatic interaction increases as the percentage of MBA increases and the optical transmittance cannot reach zero even at very high temperatures, such as in the case of NG9.

Adjustment of the solution’s pH to 7.4 facilitates deprotonation of the carboxyl groups, enhancing the electrostatic repulsive force between carboxylate anions. This explains the much shallower transition observed for both PB and TAB solutions, regardless of the crosslinker content. It has been reported that less hydrated organic ions can bind directly to the polymer’s hydrophobic moieties [39]. The positively charged organic TRIS-H^+^ and BIS-TRIS-H^+^ species in the pH = 7.4 TAB buffer can act as counterions, reducing the overall charge density of the nanogels. Therefore, a relatively more packed collapse of nanogels was observed for TAB compared to PB. When the salt content of the buffer is significantly increased, as in the case of RHB, the “salting-out” effect plays an important role in regulating the phase transition mechanism of these charged nanogels, although the repulsive electrostatic forces remain a dominating factor. In this case, unsurprisingly, steeper VPTT profiles can be observed in RHB. 

### 3.4. NPAM–APrOH–MBA Nanogels in TAB with Different pHs

**Figure 5:**
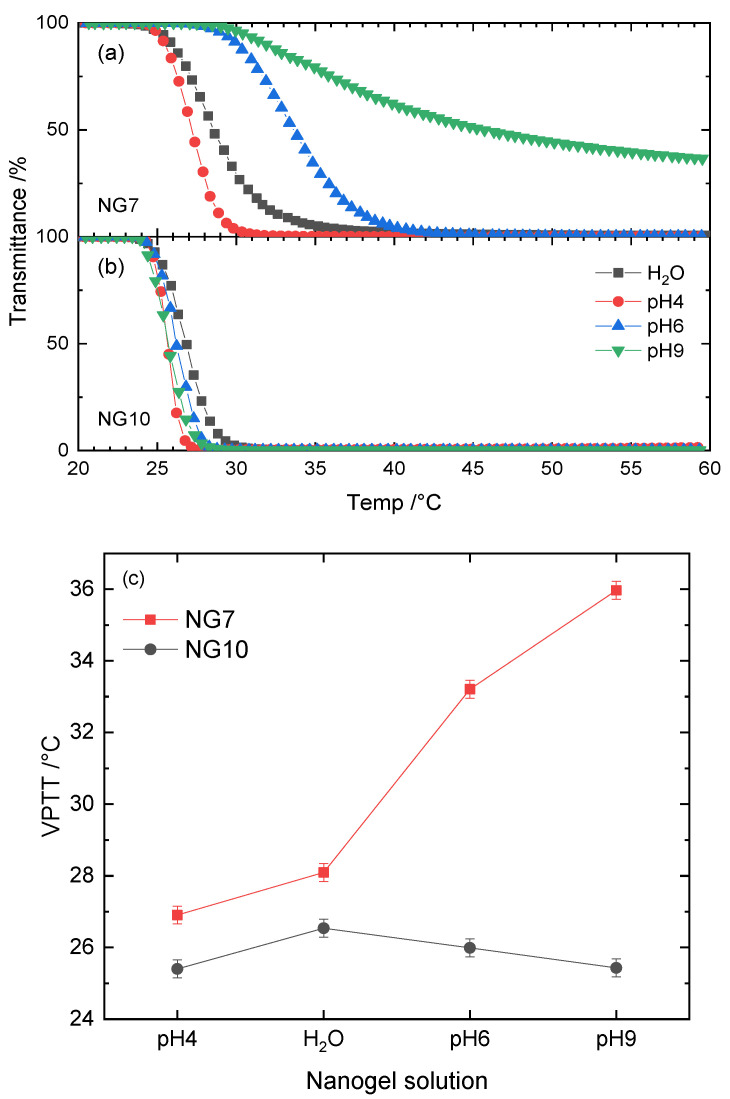
VPTT profiles of (**a**) NG7 and (**b**) NG10 in TAB buffer as a function of pH. Their corresponding VPTT values are summarized in (**c**).

The presence of the ionic functional monomer APrOH introduces a pH switch to the thermoresponsive NPAM-based nanogels. This means that NPAM–APrOH crosslinked nanogels respond differently to changes in pH. Nanogel NG7 (5%MBA, more flexible) was selected for further investigations of VPTT as a function of pH in TAB. Adjustment of the pH was obtained by the addition of diluted HCl to the buffer. To compensate for the change in ionic strength, NaCl was added accordingly (Table S3). Therefore, the variation, if any, of VPTT as a function of pH would not be due to any ionic strength effect. For the purpose of comparison, the neutral analog N10 (5%MBA–NPAM nanogel, without APrOH) was also studied under the same experimental conditions. The data are shown in Figure 5 and Figure S8 (Gaussian fits) and Table S4 (VPTT values).

The data for NPAM–APrOH nanogel NG7 show higher values of VPTT when the pH of the TAB buffer solution is changed from acidic to alkaline. The phase transition of NG7 at pH = 9 was extremely shallow and broad, resulting in an optical transmittance of 30% at 60 °C. For neutral NPAM-based nanogel NG10, the VPTT in deionized water was approximately 26 °C. In contrast to NG7, the addition of TAB marginally reduced the VPTT of NG10 and was independent of the solution’s pH.

The vast majority of carboxyl groups are protonated in the acidic pH = 4 TAB solution, since alkyl carboxyl acid is around pK_a_ 5. At this stage, the effect of surface charge of the nanogel particle is negligible. The phase transition is predominantly dictated by the strength of hydrophobic hydrations around the *n*-propyl groups. After an increase to pH = 6, electrostatic repulsions begin to take place as segments of the carboxyl groups are partially deprotonated. The phase separation of NG7 at this pH is influenced by a combination of hydrophobic interactions and electrostatic repulsions, leading to a higher VPTT. When the carboxyl groups are mostly deprotonated, as in the case of pH = 9, the repulsive electrostatic forces among the nanogel dominate. This causes the nanogel to have less of a tendency to dehydrate and aggregate, resulting in an incomplete phase transition with a higher VPTT value. On the contrary, the neutral counterpart NG10 did not show such significant variations in VPTT as a function of pH, which is expected as the nanogel particle was not functionalized with any charged groups. The slight drop in VPTT upon buffering is simply due to the “salting-out” effect.

## 4. Conclusions

In this work, we have studied the thermal characteristics of smart stimuli-responsive acrylamide-based nanogels, synthesized by high dilution radical polymerization, as a function of temperature in both deionized water, PB, TAB and RHB media. The buffers included in this study were chosen for their proximity to the physiological pH range.

The thermoresponsive properties of the acrylamide-based nanogels are highly dependent on the chemical structure of the polymeric network. For example, the VPTTs of NIPAM-based nanogels display a bell-shaped curve as a function of MBA due to the interplay between the hydrophobicity and rigidity of nanogels. A subtle change in the chemical structure of the side chains of monomers, from branched isopropyl groups to linear propyl groups leads to a reduction of the VPTT value by ~10 °C. The addition of hydrophilic comonomers (both HEAM and APrOH) not only causes the VPTT to shift to the higher value due to a much stronger affinity for water but also shallows the transition profile as a result of enhanced heterogeneity of the resultant polymeric chains.

Buffer solutions including PB, TAB and RHB facilitate the phase separation of nanogels. This “salting-out” phenomenon is caused by the disruption in hydration of the colloidal particles in the presence of ions. This phase transition is driven by intricate interplay involving the entropy of mixing, electrostatic interactions, conformational transitions, and structural rigidity.

Therefore, nanogels exhibit lower VPTTs in buffer media than in deionized water. This reduction of VPTT values strongly depends on the electrolyte concentration as well as the specificity of individual anions, e.g., their position in the Hofmeister series. Furthermore, the addition of ionizable comonomer APrOH to the NPAM monomer during polymerization grants dual thermal- and pH-responsive properties of resultant nanogels. At pH = 4, where the vast majority of carboxyl groups are protonated, the VPTT is predominantly dictated by the strength of hydrophobic hydration of the side segments. When pH > 7, the repulsive electrostatic force among the nanogel dominates. This causes the nanogel to have less of a tendency to dehydrate and aggregate, resulting in an incomplete phase transition with a higher VPTT value.

These results provide evidence that the medium where nanogels operate has a profound influence on their physicochemical properties, such as the characteristic phase transition temperature. The outcome of this work highlights the importance of the careful molecular design of stimuli-responsive nanogels and their structural tailoring for applications in complex solution environments, such as biomedical applications [44].

## Figures and Tables

**Table 1:** Chemical compositions for nanogels NG1–NG10.

NG No.	Feed Composition				
	NIPAM	HEAM	NPAM	APrOH	MBA
	mol %				
**NG1**	95				5
**NG2**	90				10
**NG3**	80				20
**NG4**	75	20			5
**NG5**	70	20			10
**NG6**	60	20			20
**NG7**			92.5	2.5	5
**NG8**			87.5	2.5	10
**NG9**			77.5	2.5	20
**NG10**			95		5

## Supplementary Materials

The following are available online at https://www.mdpi.com/2073-4360/12/11/2590/s1, Table S1. Buffer recipes and calculations of ionic strength; Table S2. VPTT values of NG1–NG9 in different media; Table S3: Recipes for TAB and NaCl solutions preparation with the same ionic strength; Table S4: VPTT values of NG7 and NG10 in TAB buffer with the addition of NaCl; Figure S1. Dynamic light scattering of nanogels NG1–NG3 in different media at 20 °C; Figure S2. Dynamic light scattering of nanogels NG4–NG6 in different media at 20 °C; Figure S3. Dynamic light scattering of nanogels NG7-NG9 in different media at 20 °C; Figure S4. Dynamic light scattering of nanogels N7 and N10 in TAB but different pH values; Figure S5. Gaussian fitting (solid lines) of the 1st derivate of VPTT profiles for NG1–NG3 in different media; Figure S6. Gaussian fitting (solid lines) of the 1st derivate of VPTT profiles for NG4–NG6 in different media; Figure S7. Gaussian fitting (solid lines) of the 1st derivate of VPTT profiles for NG7–NG9 in different media; Figure S8. Gaussian fitting (solid lines) of the 1st derivate of VPTT profiles for NG7 and NG10 in different pH TAB solution with the addition of NaCl.
