# Supplementary material for: Influence of Buffers, Ionic Strength, and pH on the Volume Phase Transition Behavior of Acrylamide-Based Nanogels
Source: Polymers (Basel). 2020 Nov 4;12(11):2590. doi: 10.3390/polym12112590 (PMC7694245; doi:10.3390/polym12112590)
Supplement: Supplementary file 1 [file polymers-12-02590-s001.pdf]

polymers

MDPI

*Supporting Information*

# Influence of Buffers, Ionic Strength, and pH on the Volume Phase Transition Behavior of Acrylamide-based Nanogels

**Harriet Louise Judah†, Pengfei Liu†, Ali Zarbakhsh* and Marina Resmini ***

Department of Chemistry, SBCS, Queen Mary University of London, Mile End Road E1 4NS, London, UK; h.l.judah@se15.qmul.ac.uk (H.L.J.); pengfei.liu@qmul.ac.uk (P.L.)

* Correspondence: a.zarbakhsh@qmul.ac.uk (A.Z.); m.resmini@qmul.ac.uk (M.R.)

† Both authors have contributed equally to the work.

## 1. Supporting Tables

**Table 1.** Buffer recipes and calculations of ionic strength.

| Buffer | pH | Component | Component concentration mM | Ionic strength mM |
|---|---|---|---|---|
| Phosphate | 7.4 | $NaH_2PO_4{\cdot}H_2O$ | 2.3 | 25 |
| | | $Na_2HPO_4$ | 7.7 | |
| TRIS Acetate | 4 | TRIS | 10 | 30 |
| | | BIS-TRIS | 10 | |
| | | $NaAc{\cdot}3H_2O$ | 10 | |
| | | HCl | 29 | |
| | 6 | TRIS | 10 | 28 |
| | | BIS-TRIS | 10 | |
| | | $NaAc{\cdot}3H_2O$ | 10 | |
| | | HCl | 19.5 | |
| | 7.4 | TRIS | 10 | 20 |
| | | BIS-TRIS | 10 | |
| | | $NaAc{\cdot}3H_2O$ | 10 | |
| | | HCl | 10 | |

| | | | | |
|---|---|---|---|---|
| | 9 | TRIS | 10 | 11 |
| | | BIS-TRIS | 10 | |
| | | NaAc·3$H_2O$ | 10 | |
| | | HCl | 1.9 | |
| Ringer HEPES | 7.4 | NaCl | 151 | 168 |
| | | KCl | 5 | |
| | | $CaCl_2$ | 2 | |
| | | $MgCl_2$ | 0.2 | |
| | | $NaHCO_3$ | 6 | |
| | | HEPES | 5 | |
| | | Glucose | 3 | |

The pKa at 25°C for $H_2PO_4^-$, $CH_3COOH$, TRIS-H, BIS-TRIS-H and $HCO_3^-$ are 7.2, 4.76, 8.07, 6.46 and 6.30, respectively. HEPES is a zwitterionic sulfonic acid buffering agent. When calculating the ionic strength of Ringer HEPES buffer, we ignored the contribution of HEPES. This has a negligible effect onto the final ionic strength value because of the presence of the excess amount of NaCl in the Ringer HEPES buffer.

**Table 2.** VPTT values of NG1-NG9 in different media.

| Nanogels No. | Chemical composition | | | | | VPTT (± 0.25 °C) | | | |
|---|---|---|---|---|---|---|---|---|---|
| | NIPAM | NPAM | HEAM | APrOH | MBA | $H_2O$ | PB* | TAB* | RHB* |
| | mol% monomer | | | | mol% XL | °C | | | |
| NG1 | 95 | 0 | 0 | 0 | 5 | 36.6 | 35.1 | 35.5 | 33.5 |
| NG2 | 90 | 0 | 0 | 0 | 10 | 39.0 | 37.0 | 37.5 | 35.9 |
| NG3 | 80 | 0 | 0 | 0 | 20 | 39.7 | 36.9 | 37.5 | 37.0 |
| NG4 | 75 | 0 | 20 | 0 | 5 | 55.4 | 53.2 | 54.1 | 51.5 |
| NG5 | 70 | 0 | 20 | 0 | 10 | 57.5 | 55.4 | 56.5 | 54.2 |
| NG6 | 60 | 0 | 20 | 0 | 20 | 57.0 | 54.9 | 56.6 | 55.7 |
| NG7 | 0 | 92.5 | 0 | 2.5 | 5 | 28.1 | 36.6 | 37.9 | 36.5 |
| NG8 | 0 | 87.5 | 0 | 2.5 | 10 | 31.8 | 40.3 | 39.4 | 37.9 |
| NG9 | 0 | 77.5 | 0 | 2.5 | 20 | 39.3 | n.d.# | n.d. | 40.0 |

* pH=7.4 for all buffers; # n.d. means not determined.

**Table 3.** Recipes for TAB and NaCl solutions preparation with the same ionic strength.

| pH of TAB | ionic strength | addition of NaCl |
|---|---|---|
| | mM | mM |
| 4 | 30 | 0 |
| 6 | 28 | 2 |
| 9 | 11 | 19 |

**Table 4.** VPTT values of NG7 and NG10 in TAB buffer with addition of NaCl.

| Nanogels No. | NPAM | APrOH | MBA | VPTT | | | |
|---|---|---|---|---|---|---|---|
| | | | | $H_2O$ | TAB + NaCl | | |
| | | | | | pH4 | pH6 | pH9 |
| | mol% monomer | | mol% XL | °C | | | |
| NG7 | 92.5 | 2.5 | 5 | 28.1 | 26.9 | 33.2 | 36.0 |
| NG10 | 95 | 0 | 5 | 26.5 | 25.4 | 26.0 | 25.4 |

## 2. Supporting Figures

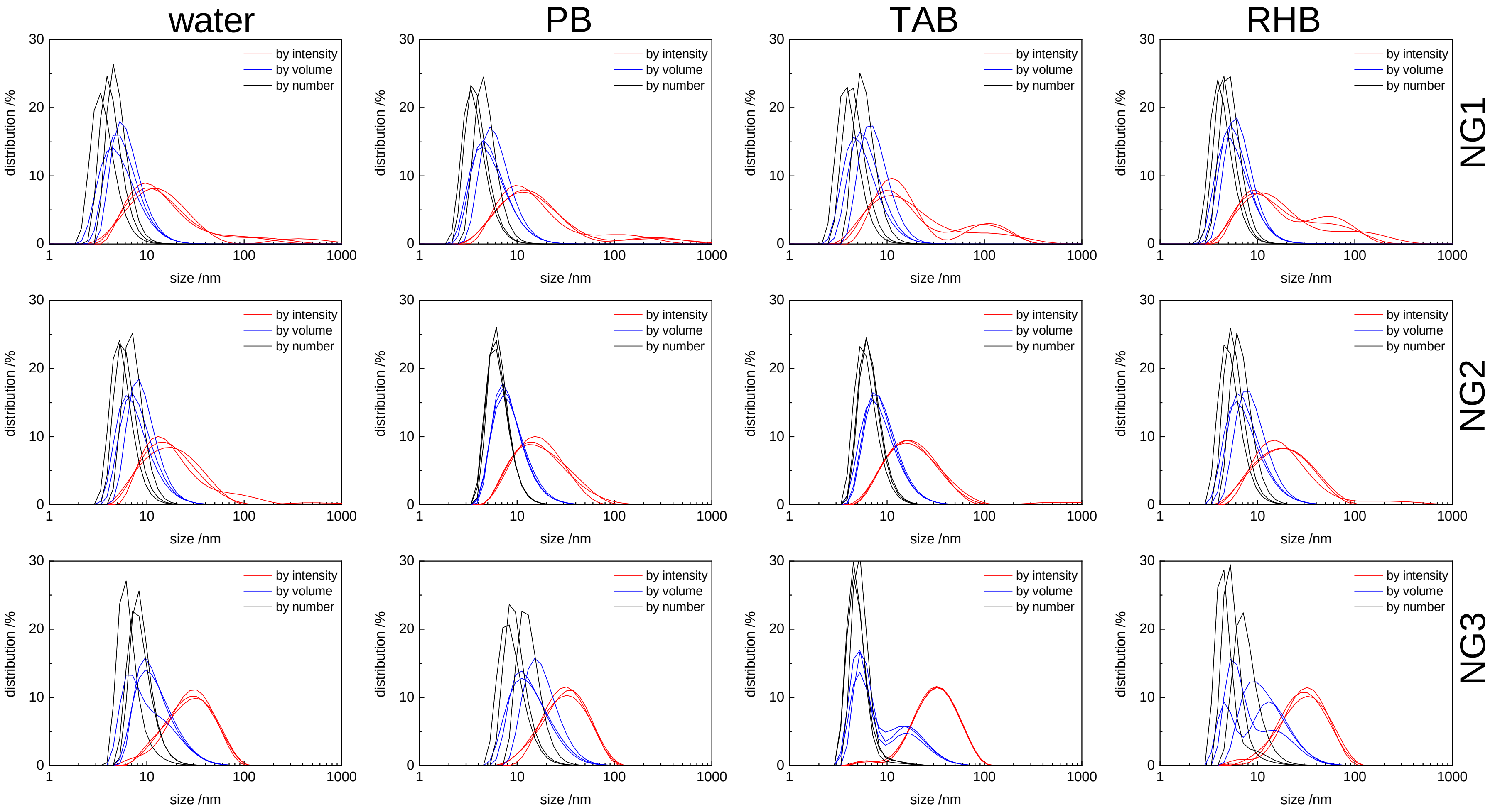


**Figure 1.** Dynamic light scattering of nanogels **NG1–NG3** in different media at 20 °C.

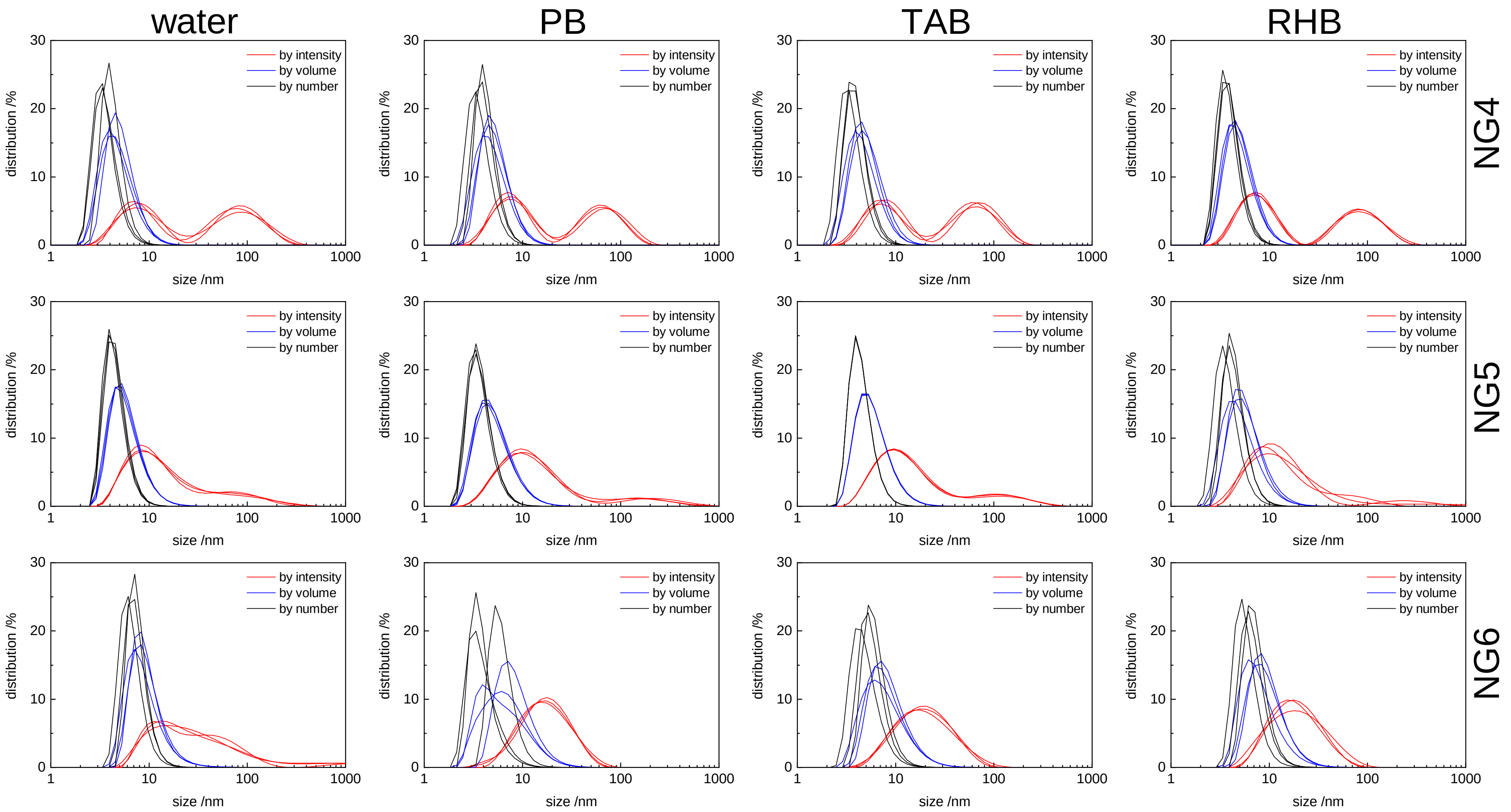


**Figure 2.** Dynamic light scattering of nanogels **NG4–NG6** in different media at 20 °C.

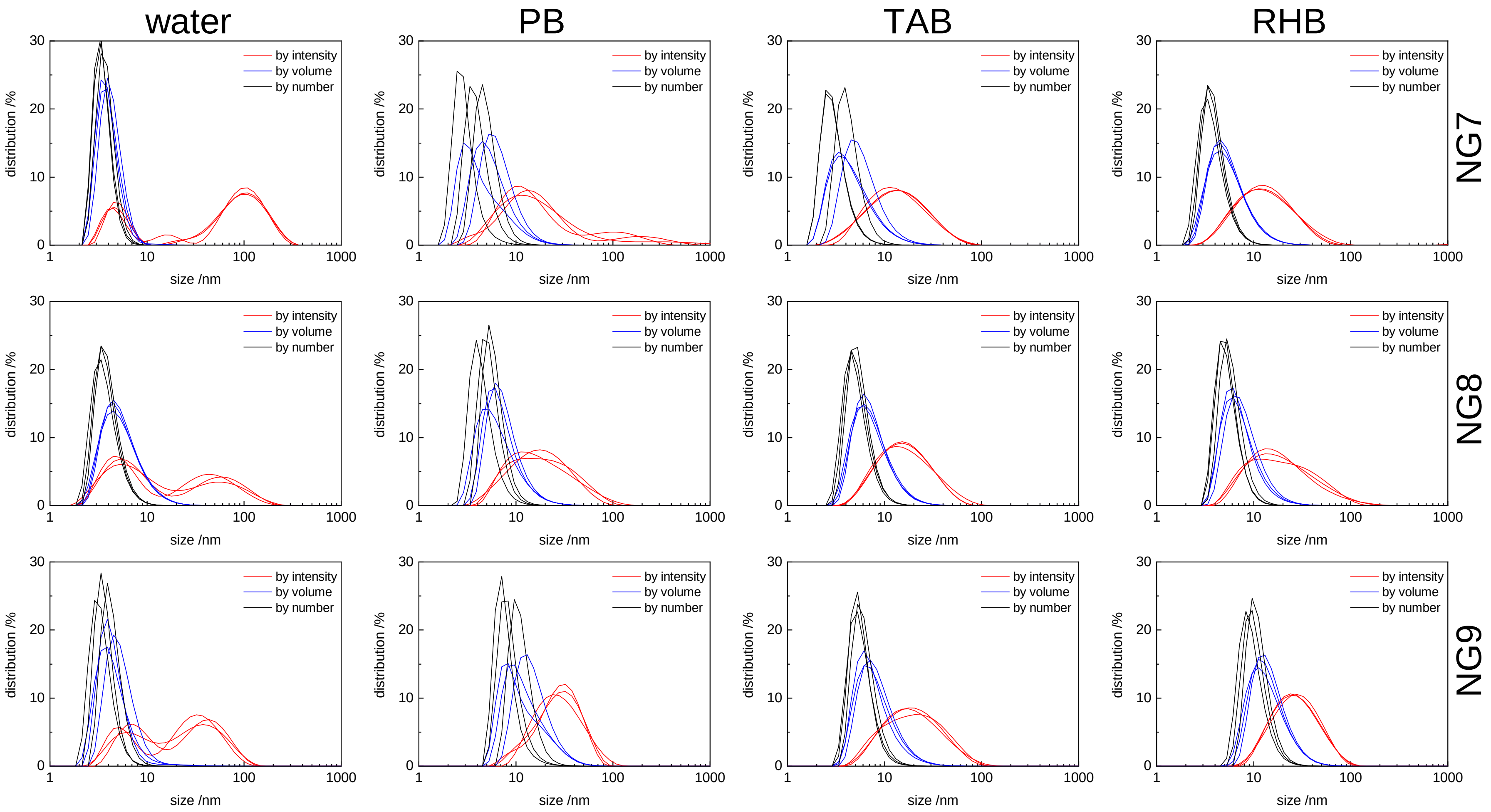


**Figure 3.** Dynamic light scattering of nanogels **NG7–NG9** in different media at 20 °C.

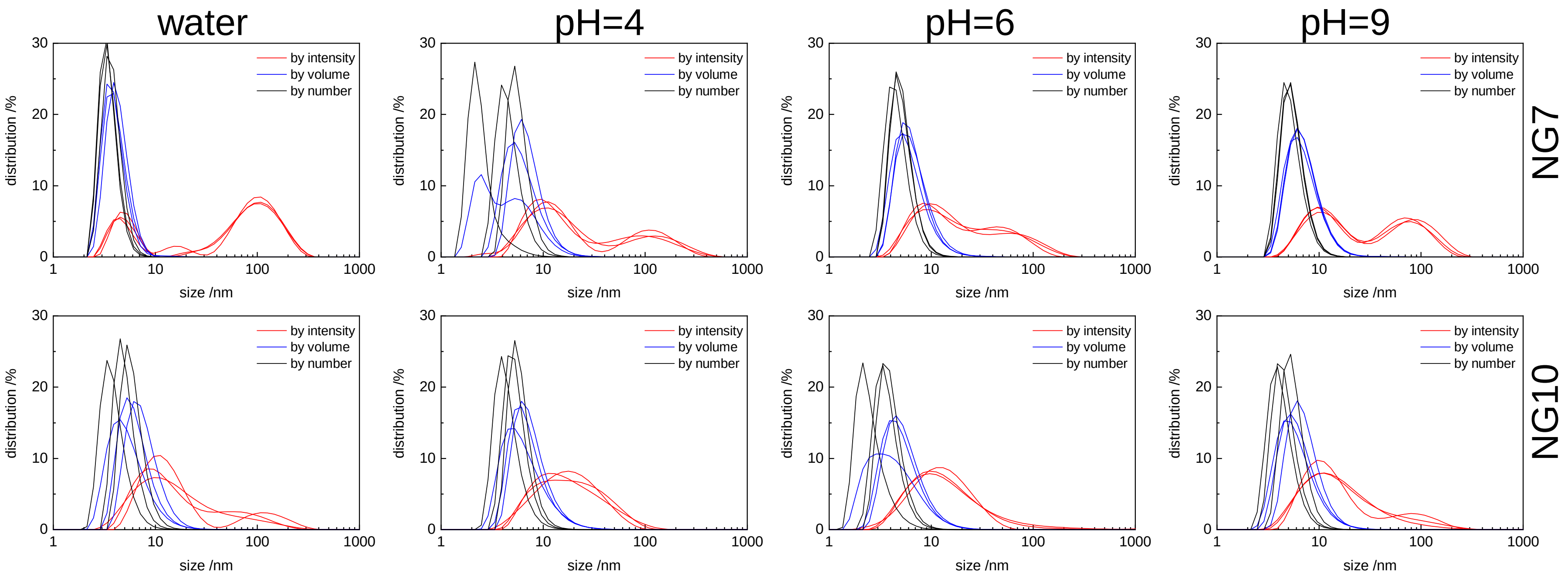


**Figure 4.** Dynamic light scattering of nanogels **N7 and N10** in TAB but different pH values. For nanogels in pH=4 TAB buffer, they were measured at 15°C because aggregation of nanogels occurred even at 20°C (data not shown).

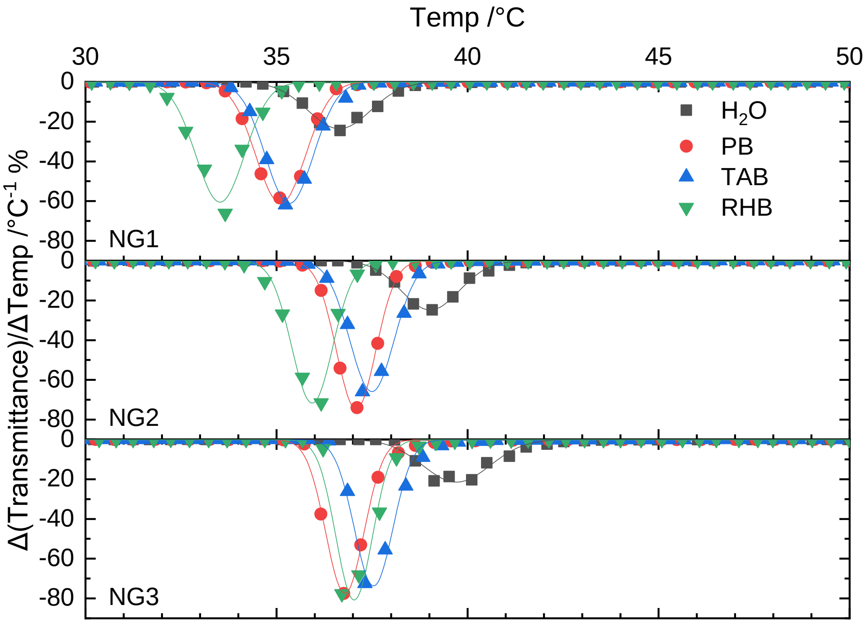


**Figure 5.** Gaussian fitting (solid lines) of 1st derivate of VPTT profiles for NG1–NG3 in different media.

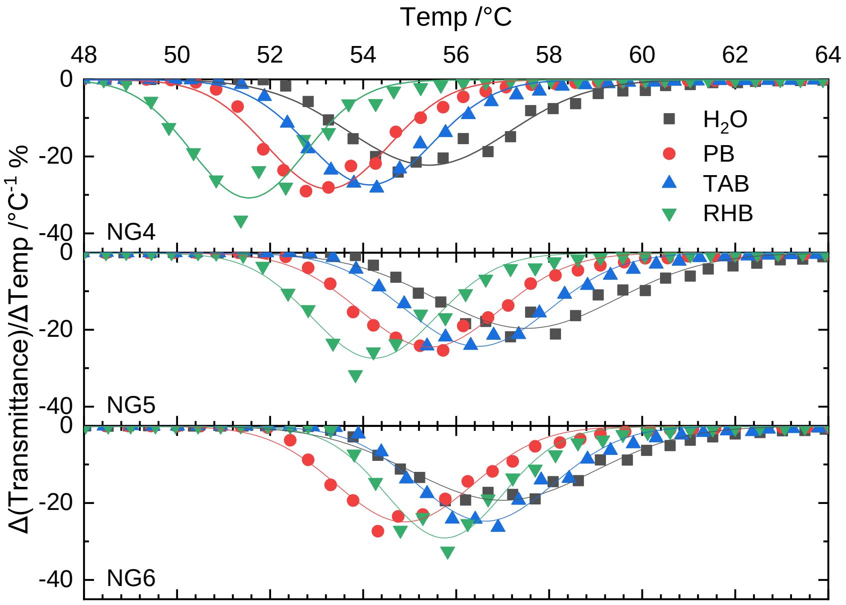


**Figure 6.** Gaussian fitting (solid lines) of 1st derivate of VPTT profiles for NG4–NG6 in different media.

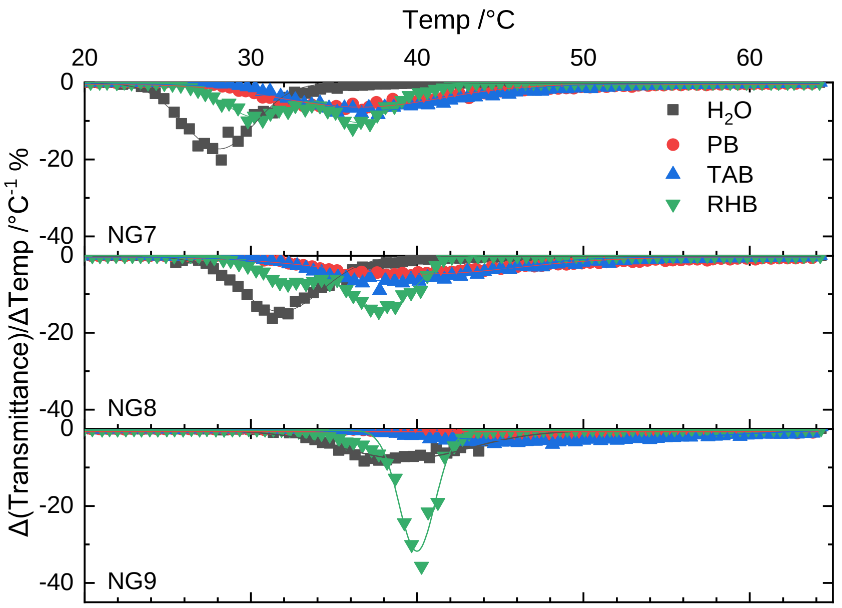


**Figure 7.** Gaussian fitting (solid lines) of 1st derivate of VPTT profiles for NG7–NG9 in different media.

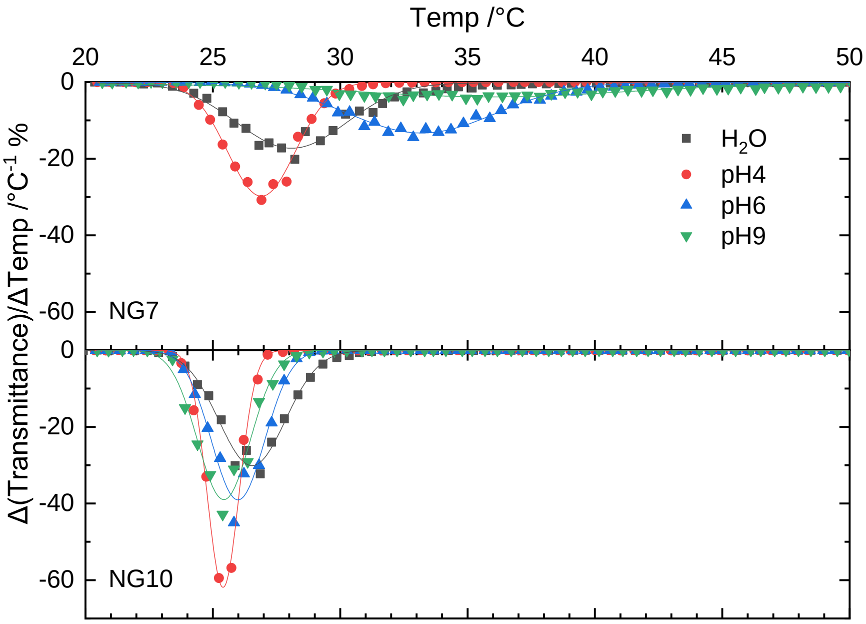


**Figure 8.** Gaussian fitting (solid lines) of 1st derivate of VPTT profiles for NG7 and NG10 in different pH TAB solution with the addition of NaCl.

© 2020 by the authors. Licensee MDPI, Basel, Switzerland. This article is an open access article distributed under the terms and conditions of the Creative Commons Attribution (CC BY) license (http://creativecommons.org/licenses/by/4.0/).
